# Evaluation of the SD Bioline TB Ag MPT64 test for identification of *Mycobacterium tuberculosis* complex from liquid cultures in Southwestern Uganda

**DOI:** 10.4102/ajlm.v6i2.383

**Published:** 2017-03-31

**Authors:** Patrick Orikiriza, Dan Nyehangane, Daniel Atwine, John J. Kisakye, Kennedy Kassaza, Juliet-Mwanga Amumpaire, Yap Boum

**Affiliations:** 1 Epicentre Mbarara Research Centre, Mbarara, Uganda; 2 Department of Microbiology, Faculty of Medicine, Mbarara University of Science and Technology, Mbarara, Uganda; 3 Department of Biological Sciences, College of Natural Sciences, Makerere University, Kampala, Uganda

## Abstract

**Background:**

To confirm presence of *Mycobacterium tuberculosis* complex, some tuberculosis culture laboratories still rely on para-nitrobenzoic acid (PNB), a traditional technique that requires sub-culturing of clinical isolates and two to three weeks to give results. Rapid identification tests have improved turnaround times for mycobacterial culture results. Considering the challenges of the PNB method, we assessed the performance of the SD Bioline TB Ag MPT64 assay by using PNB as gold standard to detect *M. tuberculosis* complex from acid-fast bacilli (AFB) positive cultures.

**Objectives:**

The aim of this study was to determine the sensitivity, specificity and turnaround time of the SD MPT64 assay for identification of *M. tuberculosis* complex, in a setting with high prevalence of tuberculosis and HIV.

**Methods:**

A convenience sample of 690 patients, with tuberculosis symptoms, was enrolled at Epicentre Mbarara Research Centre between April 2010 and June 2011. The samples were decontaminated using NALC-NaOH and re-suspended sediments inoculated in Mycobacterium Growth Indicator Tubes (MGIT) media, then incubated at 37 °C for a maximum of eight weeks. A random sample of 50 known negative cultures and 50 non-tuberculous mycobacteria isolates were tested for specificity, while sensitivity was based on AFB positivity. The time required from positive culture to reporting of results was also assessed with PNB used as the gold standard.

**Results:**

Of the 138 cultures that were AFB-positive, the sensitivity of the SD MPT64 assay was 100.0% [95% CI: 97.3 – 100] and specificity was 100.0% (95% CI, 96.4 – 100). The median time from a specimen receipt to confirmation of strain was 10 days [IQR: 8–12] with SD MPT64 and 24 days [IQR: 22–26] with PNB.

**Conclusion:**

The SD MPT64 assay is comparable to PNB for identification of *M. tuberculosis* complex and reduces the time to detection.

## Introduction

Proper diagnosis is the first step toward better management and prevention of tuberculosis transmission. The Stop TB Partnership has recently described the actions and resources needed to end tuberculosis in the world by 2030^1^. The world is now focusing on a ‘paradigm shift’ that will see countries improve case finding and decrease tuberculosis incidence rates by at least 10% annually. In order to achieve this global plan to end tuberculosis, all healthcare practitioners will be required to ensure that 90% of vulnerable groups are screened, 90% of those diagnosed are started on treatment and 90% are successfully treated. As such, one of the global priorities is on diagnosis.^1^ Discovery, development and rapid uptake of new tools and interventions have been highlighted as major requirements for the success of this plan.

Currently, significant success has been noted with the integration of the Xpert^®^ MTB/RIF (Cepheid, United States) assay into clinical practice.^2^ While this tool has the advantage of detecting *Mycobacterium tuberculosis* complex and mutations associated with rifampicin resistance, within approximately two hours^2^, challenges exist in low-resource settings related to instrument breakdown, inconsistent electric power supply, delayed maintenance, irregular supply of cartridges, limited machine capacity (four tests every two hours) and errors.^2^ Despite the new tool, approximately three million tuberculosis cases annually are not diagnosed,^1^ thus causing a public health challenge.

Tuberculosis and HIV form a deadly synergy,^3^ yet the number of bacilli in the sputum samples of co-infected patients is usually low. As a result, countries including Uganda have adopted national tuberculosis programme guidelines that prioritise the use of the Xpert^®^ MTB/RIF assay on sputum from vulnerable groups such as children and on acid-fast bacilli (AFB)-smear-negative, HIV-positive patients with signs and symptoms of tuberculosis.^4^ However, the majority of persons with presumptive tuberculosis do not necessarily know their HIV status and may not be eligible for the Xpert^®^ MTB/RIF assay.

Bacteriological confirmation of tuberculosis by culture isolation currently remains the diagnostic reference standard recommended by the World Health Organization.^5^ However, even with a positive liquid culture there is need to differentiate *M. tuberculosis* complex and non-tuberculous mycobacteria (NTM). Although this can easily be achieved by the current molecular methods such as line probe assays, these are complex and bear high infrastructural and human resource requirements.^6^

Traditional methods, such as the use of para-nitrobenzoic acid (PNB) on Ziehl-Neelsen (ZN) positive cultures, are simple, but require pure isolates, which delays results (2–3 weeks).^6, 7^ This delay impacts clinical management of the patient and potentially prolongs transmission among contacts of tuberculosis patients. There are additional costs associated with incubation requirements, such as staff time, space in the incubator, electricity and other factors that are not always considered.^7^

Recently, rapid methods of identifying *M. tuberculosis* complex from AFB-positive cultures have been developed. These rely on chromatographic detection of MPT64, a protein that is produced by *M. tuberculosis* complex during its metabolism in cultures.^8^ Among these, the two commonly-available methods include the Capilia TB-Neo assay (Tauns Laboratories, Inc., Numazu, Japan) and the SD Bioline TB Ag MPT64 assay (Standard Diagnostics, Yongin-si, Gyeonggi-do, Republic of Korea; hereafter, SD MPT64 [assay]). These assays have the advantage of being inexpensive, easy to use and readily available, even in low-resource settings.^9^ They are easily stored at room temperature and allow for results from positive cultures within 15 minutes.^10^ Available data from evaluation of the Capilia TB-Neo assay indicate that it has excellent sensitivity and specificity.^9,11,12^ Few studies have been done on the SD MPT64 assay in field settings.^13,14,15^ Nevertheless, from the results of these studies, there is general agreement that these methods are suitable replacements for the traditional methods.

The aim of this study was to determine the sensitivity, specificity and turnaround time of the SD MPT64 assay for identification of *M. tuberculosis* complex, in a setting with high prevalence of tuberculosis and HIV.

## Methods

### Ethical considerations

The samples were obtained from patients enrolled at the Epicentre Mbarara Research Centre with approval from the Faculty Research and Ethics committee and the Institutional Review Board at Mbarara University of Science and Technology, as well as the Uganda National Council for Sciences and Technology. All patients signed an informed consent form to participate in the main study and allow further testing on the samples and isolates.

### Study design

Samples for this cross-sectional study were obtained from patients with signs and symptoms of pulmonary tuberculosis according to World Health Organization guidelines.^16^ We enrolled 690 patients at the Epicentre Mbarara Research Centre, Mbarara, Uganda, between April 2010 and June 2011 in a separate study to assess the utility of colorimetric methods to detect *M. tuberculosis* complex in patients with suspected tuberculosis.^17^ Patients were eligible if they reported a cough for more than two weeks, were at least 15 years of age and signed an informed consent. After consenting, patients’ samples were collected, stored in a cool box and then transported immediately to the laboratory. They were decontaminated using the N-acetyl L-cysteine (NALC)-NaOH procedure.^18^ The concentrated sediments were homogenised with phosphate buffer, inoculated in Mycobacterium Growth Indicator Tubes (MGIT) (BD, Franklin Lakes, New Jersey, United States) medium and then incubated at 37 °C for up to eight weeks. MGIT cultures were read daily on a manual reader until growth was detected. To check for the presence of AFB all positive cultures were tested by Ziehl-Neelsen (ZN) smear microscopy; to check for contamination, positive cultures were tested by blood agar. The Epicentre Laboratory participates in an external quality assurance scheme for culture with the National Health Laboratory Services, South Africa.

For this study, a convenience sample of 138 AFB-positive cultures was considered for testing with SD MPT64 and PNB. The two tests were performed immediately on the isolate according to the manufacturer’s recommendations and laboratory protocol. If AFB was present together with contamination, the isolate was re-decontaminated with NaOH, sub-cultured in a fresh culture tube and monitored for pure growth before repeating PNB.

**FIGURE 1:**
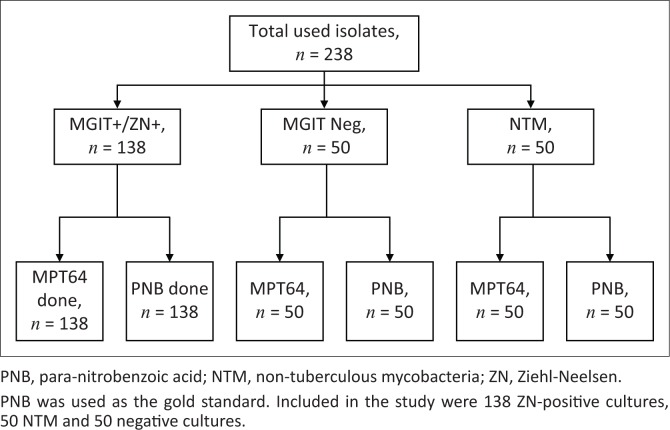
Study Profile.

To determine specificity, we randomly selected 50 known-negative MGIT samples from the study, which were tested independently with SD MPT64 and PNB. To confirm ability of the test to rule out NTMs, an additional sample of 50 known isolates of *Mycobacterium fortuitum* were sub-cultured into MGIT using a standard inoculum of McFarland 0.5. These were monitored daily until positivity was detected by the MGIT instrument. We performed the SD MPT64 assay and PNB on each of them as described above for the MGIT-negative cultures (Figure 1).

The PNB media was prepared locally, stored between 2 °C – 8 °C and quality controlled according to our standard operating procedures. The test required two Lowenstein Jensen slants one with and another without PNB reagent, the latter serving as negative control. Both slants were inoculated with 500 µL of standard inoculum, incubated at 37 °C and read weekly until colonial growth was observed. Identification of *M. tuberculosis* complex was made based on presence of growth in the control tube (without PNB), with no growth in the tube with PNB.

Manufacturer instructions were followed in performing the SD MPT64 assay. In brief, 100 µL of mixed MGIT culture was added directly to the test cartridge and allowed to flow chromatographically for 15 minutes. A positive result was indicated by a red band on the test window in addition to the control band. Data were collected using case report forms and double entered using Voozanoo software version 2 (EpiConcept, Paris, France). Turnaround time was calculated by measuring the time taken from specimen receipt to reporting of a positive SD MPT64 or PNB assay. Ease of use of the techniques was determined through a questionnaire that was filled out by all technicians performing the tests.

### Statistical analyses

Statistical analysis was performed using Stata SE v.11 software (College Station, Texas, United States, 2009). We considered a sample positive with *M. tuberculosis* complex when confirmed by the PNB gold standard and negative if not confirmed positive for *M. tuberculosis* complex by the gold standard. For SD MPT64, performance was calculated by estimating the sensitivity, specificity and 95% confidence interval.

## Results

The median age (years) and interquartile range (IQR) was 38 (30–48) with a gender ratio (male/female) of 49/51 and HIV positivity of 58.6%. Of the 138 AFB-positive MGIT cultures among the 690 patients included in the study, 136 cultures (98.6%) were confirmed positive for *M. tuberculosis* complex by both SD MPT64 and PNB, and two as NTM (Table 1). This gave a sensitivity of 100% [95% CI: 97.3–100]. All the known negative cultures and NTM were reported as negative for *M. tuberculosis* complex, giving a specificity of 100.0% [95% CI: 96.4–100], respectively.

**TABLE 1:** Cross tabulation of SD MPT64 assay and para-nitrobenzoic acid.

SD MPT64 assay	Reference standard (PNB)			
	Negative	*M. tuberculosis*	NTM	Total
**Positives**				
Negative	0	0	0	0
*M. tuberculosis*	0	136	0	136
NTM	0	0	2	2
**Negatives**				
Negative	50	0	0	50
*M. tuberculosis*	0	0	0	0
NTM	0	0	0	0
**NTM**				
Negative	0	0	0	0
*M. tuberculosis*	0	0	0	0
NTM	0	0	50	50

PNB, para-nitrobenzoic acid; NTM, non-tuberculous mycobacteria; ZN, Ziehl-Neelsen.

The median time from specimen receipt to confirmed identification of *M. tuberculosis* complex was 10 days [IQR: 8–12 days] with SD MPT64 and 24 days [IQR: 22–26] with PNB. All the technicians who performed the laboratory tests reported that the SD MPT64 assay was easy to use and did not require additional training other than the standard operating procedure. This was not the case with the PNB method.

## Discussion

Few studies have evaluated the performance of the SD MPT64 assay in field settings with high tuberculosis and HIV burdens. This study confirms prior evaluations and increases the evidence that the test has excellent sensitivity and specificity in identifying *M. tuberculosis* complex using PNB as a gold standard, in a Ugandan field setting where there is high tuberculosis and HIV co-infection. There have been previous evaluations in various countries using different gold standards. All the investigations have shown that the technique has high sensitivity and specificity. One study used reference bacterial strains and *Mycobacterium bovis* field isolates from animals and found a sensitivity of 96.5% [95% CI: 91.2–99.0] and specificity of 100% [95% CI: 96.7–100].^19^ The same group also found a high positive predictive value of 100% [95% CI: 96.7–100], and a negative predictive value of 92.9% [95% CI: 82.7–98.0].

The SD MPT64 assay was evaluated on a large number of clinical isolates in India and performed with 100.0% sensitivity and specificity.^9^ Another recent study in India used isolates from extra-pulmonary and NTM samples and found SD MPT64 to have a 100.0% sensitivity and specificity compared with conventional tests such as niacin, nitrate reduction and PNB.^13^ Although the sample size was small, they demonstrated the accuracy, cost effectiveness and early identification of *M. tuberculosis* complex in patients with paucibacillary extra-pulmonary tuberculosis.

The performance of the Capilia TB-Neo assay has been evaluated extensively. One study evaluated its performance using reference strains of *M. tuberculosis* complex, NTM and other non-related bacteria. They used nucleic acid assays in addition to the Capilia TB-Neo and SD MPT64 assays. The Capilia TB-Neo assay had 99.6% sensitivity and 100% specificity, unlike the SD MPT64 assay which had several false positive results with the NTM that were attributed to a high concentration of the bacterial antigen^20^. This poor specificity, however, was not observed in our study possibly due to only one NTM species evaluated. In Uganda, a study done in Kampala city provided evidence that the Capilia TB-Neo assay can be used to identify *M. tuberculosis* complex successfully from blood cultures.^12^ They found an overall sensitivity of 98.4% and overall specificity of 97.6% compared to polymerase chain reaction, which supports its use for blood culture isolates.

### Limitations

This study was nested within a colorimetric study and thus relied on study design, population selection and sample size of the primary study. The lack of other NTM strains limited exploration of false positivity as reported in other studies.

### Recommendations

Basing on our findings, we recommend that the SD Bioline TB Ag MPT64 test be used in place of PNB for identification of *M. tuberculosis* complex from liquid cultures.

### Conclusion

The SD Bioline MPT64 assay has good sensitivity and specificity for rapid discrimination between *M. tuberculosis* complex and NTM in clinical isolates. It also has a short turnaround time in confirming *M. tuberculosis* complex in AFB-positive cultures. In addition, the technicians reported that the test is easy to perform.
